# Dysfunctional Early Processing of Facial Expressions in Hazardous Drinkers: Evidence from an ERP Study

**DOI:** 10.1038/s41598-017-13935-7

**Published:** 2017-10-17

**Authors:** Hui Zhang, Yi Jin, John S. Y. Chan, Feng-Chi Yang, Fang Cui

**Affiliations:** 1 School of Health Management and Education, Capital Medical University, Beijing, China; 2 State Key Laboratory of Cognitive Neuroscience and Learning, Beijing Normal University, Beijing, China; 3 Shenzhen Key Laboratory of Affective and Social Cognitive Science, Shenzhen University, Shenzhen, China

## Abstract

Chronic alcohol intoxication impairs multiple cognitive functions. According to the dual system model (DSM), the development of alcohol dependence (AD) involves the imbalance between the automatic-affective system and the reflective system. However, the cognitive functions of non-AD hazardous drinkers (HDs) remain unclear. The present study aimed to explore how the HDs process facial expressions differently from the healthy subjects. Sixteen HDs and seventeen control subjects (CSs) completed an emotional working memory (WM) task while the electroencephalogram (EEG) was recorded. We found that there was no significant group difference in behavioral performance between the two groups. In the ERP data, relative to the CSs, the HDs showed delayed latencies of P1 and N170. Moreover, the CSs showed significant differences between the amplitudes of neural/fear and disgust expressions while these differences were insignificant in the HDs. The current results suggest that the main deficits in the processing of facial expression in HDs existed in the early automatic-affective system instead of in the reflective system.

## Introduction

Previous studies suggested that chronic alcohol consumption impairs cognitive functions. For instance, alcohol-dependent (AD) participants not only tended to orient to task-irrelevant stimuli automatically compared with the CSs but also had difficulty in shifting attention back to the task from the distractor^1^. Impaired inhibitory control ability was observed in the ADs indexed by a reduced P3 amplitude in the go/no-go task^2^. Using the modified Attention Network Test (ANT), Maurage *et al*. (2014) found that the ADs have a deficient executive attention network^3^, which involves deficits in both of the top-down control of attention and the inhibitory processing of irrelevant stimuli^4^.

The frontal lobe is particularly vulnerable to alcoholism^5^. Previous studies have demonstrated that frontal lobe shrinkage^6^, reduced prefrontal cortex (PFC) activation^7^, and reduced frontal lobe metabolism^8,9^ in chronic alcoholics. One of the important cognitive abilities supported by the frontal lobe is working memory (WM)^10^, which is impaired in the Ads, especially in the early abstinence period^11^.

In addition to the frontal lobe, chronic alcohol intoxication also influences the brain regions associated with emotional processing, such as the limbic circuits including the hippocampus, thalamus, cingulate cortex^12^ and the amygdala^13^, thereby contributing to the deficits in emotional abilities, such as empathy^14^, irony understanding^15^, and the decoding of emotional stimuli such as emotional facial expressions^16^ or emotional voices^17^. Chronic alcoholics showed an increased fear response to all emotional faces^18^ and they also showed the tendency to overestimate the intensity of portrayed emotions^19^.

Nevertheless, the studies aforementioned were all focusing on ADs, and the generalization of these results to those with a less severe alcohol consumption history is not justified. According to the continuum hypothesis, compared to the ADs, the hazardous effects of alcohol intoxication on the cognitive system of non-AD people should be similar in “quality” but less in “quantity”^20^. That is, the non-AD hazardous drinkers (HDs) may also have an abnormal response to emotional cues and impaired working memory performance as the ADs do, but to a lesser extent.

The current study is aimed to explore the cognitive functions in HDs. An emotional working memory (WM) task was used to exam how the participants process and encode the emotional stimulus in the WM. For the purpose of examining whether the cognitive functions related to emotional WM are intact in the HDs, we recruited a group of participants with no history of hazardous drinking as the control subjects (CSs). According to the literature and the current data, we mainly focused on four components. P1, a positive wave with the maximal peak occurring at around 100 ms after the stimulus onset, reflecting the response to low-level visual cues during early perceptual stage^21,22^, is selected for analysis. N170 is also considered as it is associated with structural representations of a stimulus for subsequent face recognition stages^23^. With a temporal-occipital distribution on the scalp, the underlying sources of the N170 were localized at multiple areas in the parieto–temporal–occipital network^24^, some of these sources were preferentially or selectively activated to facial expressions as observed in neuroimaging studies^25^. Besides, some attention related components were also analyzed. P2, a frontally maximal, positive-going component peaking around 200 ms, reflects an implicit, higher-order perceptual process when a stimulus is being compared with mental representations in WM^26^. P2 is related to the bottom-up processing of the stimulus^27^, thus could be an indicator of the automatic processing of emotional stimuli. P3 is a positive deflection that often occurs between 300 ms to 500 ms after the onset of stimuli. This component reflectsattentionalfunctions^28^, and is maximal at the posterior sites like CPz and Pz^29^. It is associated with target detection, WM operations, and other cognitive processes^28^. The P3 observed in the current study should reflect conscious evaluation processes driven by top-down control for memory encoding^30^. According to the previous research of ADs, we hypnotized that the HDs may have abnormal P2 activity triggered by the bottom-up response to emotional stimulus compared with the CSs. Meanwhile, the HDs may have reduced P3 activity together with poorer WM performance relative to the CSs due to the dysfunctional WM ability. Besides, because some early perceptual abnormalities have also been reported in the ADs^31,32^, the HDs may also exhibit a reduced amplitudes or longer latencies of P1 or N170.

## Method

### Participants

Sixteen HDs (all male, age 26.63 ± 4.87y), who scored 8 or above^33^ in the Alcohol Use Disorders Identification Test (AUDIT)^34^, participated in this study. They were matched with another seventeen male CSs who had AUDIT scores less than 8 (age 25.35 ± 4.05y). All the participants had a normal or corrected-to-normal vision and were free of any history of psychiatric or neurological disorders or other substance abuses. They were assessed with the State-Trait Anxiety Inventory (S-TAI)^35^ and the Beck Depression Inventory (BDI)^36^ to control the levels of anxiety and depression. The mean scores and standard deviation on BDI/S-TAI/AUDIT of the two groups were reported in Table 1. T-tests revealed a significant group difference in AUDIT (*t* = −12.86, *p* < 0.001) while no significant differences in BDI, TAI or SAI (*p*s > 0.12), thus confirming the reasonable matching between the groups. All of the participants abstained from alcohol consumption for at least 2 weeks before the experiment. They were paid 80RMB for their participation. The experiment was conducted in accordance with the Declaration of Helsinki and was approved by the Medical Ethical Committee of the Psychology School of Beijing Normal University, China. Written informed consent was obtained from each participant.

**Table 1:** Psychological Measures for Hazardous Drinkers (HDs) and Control Subjects (CSs): Mean (SD).

	HDs (N = 16)	CSs (N = 17)	*t*
AUDIT	12.75 (3.34)	2.06 (2.08)	−11.13^a^
BDI	5.00 (3.79)	4.00 (3.50)	−0.79
TAI	33.44 (6.05)	32.76 (6.49)	−0.31
SAI	30.00 (6.23)	30.82 (6.61)	−0.37

Note. AUDIT = Alcohol Use Disorders Identification Test; BDI = Beck Depression Inventory; TAI = Trait Anxiety Inventory; SAI = State Anxiety Inventory. ^a^ *p* < 0.001

### Material

The pictures of facial expressions were selected from the Chinese Affective Picture System (CAPS)^37^. Each expressed one of the three basic emotions (20 faces for each emotion, displayed by 10 actors and 10 actresses): fear, disgust, and neutral. 15 participants who did not participate in the later EEG task rated the valence and arousal levels on 9-point-scales for each of the pictures. The result of the rating showed that for the arousal level, both of the fear (5.92) and disgust (5.81) expressions were more arousing than the neutral ones (3.21, *p*s < 0.001) while the difference between fear and disgust was insignificant. For the valence, both of the fear (6.70) and disgust (6.71) expressions were more negative than the neutral faces (4.40, *p*s < 0.001) while the difference between the two negative expressions were insignificant.

### Procedure

**Figure 1:**
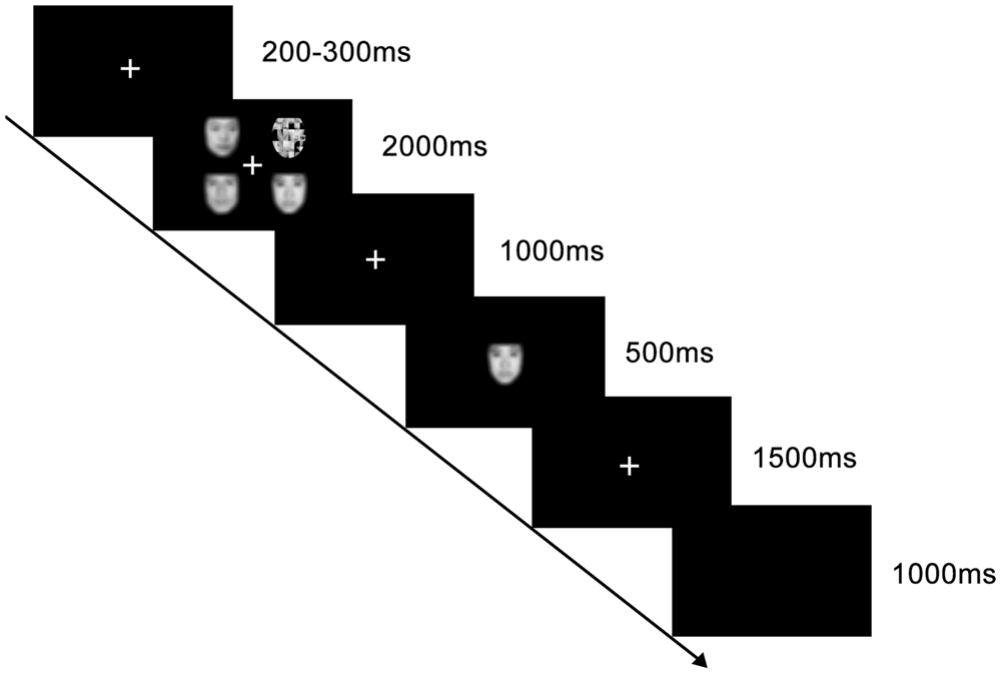
Illustration of a trial in the emotional working memory (WM) task (*i.e*., neutral trail in high WM load, the correct response being “yes”) (Notice: the faces in the pictures were blurred for the privacy of the actor/actress appeared in it. In real experiment, the faces are clear and recognizable).

The emotional WM task was adopted from the Morgan *et al*.^38^. Participants were instructed to memorize faces under low/high memory loads (*i.e*., one face vs three faces). Each face (64 × 74 pixels) was gray-scaled, presented against a black background. Each trial began with the presentation of a white fixation for a random period of time (200~300 ms). Then one emotional face and three scrambled faces (Low WM load), or three emotional faces and one scrambled face (High WM load) appeared for 2,000 ms (visual angle 7.52° × 6.88°). After a 1,000 ms blank interval, a single face would show in the center of the screen as a probe (visual angle 3.22° × 3.44°) for 500 ms. Then a blank would last for maximum1, 500 ms till a response was given by the participant. The participants were required to judge whether the probe had been presented in the WM load as quickly and accurately as possible by pressing the corresponding key on a keyboard. The responding keys were counterbalanced across participants)(Fig. 1).

The present experiment consisted of 384 trials, divided into six blocks with short breaks between two consecutive blocks. The faces showed the same emotion (*i.e*., neutral, fear or disgust, there were two blocks for each emotion) and two levels of WM loads were randomized within each block. Each block lasted about 7 minutes and the whole experiment lasted about1 hour. The display of stimuli and the acquisition of behavioral data were performed using E-Prime 2.0 software (Psychology Software Tools). During the task, participants sat comfortably in an electrically shielded room approximately 100 cm from a 15-inch color computer screen. Before the experiment participants were given a training session, consisting of 16 randomly selected neutral trials to ensure they fully understood the task. EEG was recorded during the task.

### EEG Acquisition and Processing

EEG data were collected at a sampling rate of 500 Hz from 64 locations of the international 10/20 system^39^ using Brain Products (Brain Products GmbH, Germany). All impedances were kept under 10 kΩ. A pair of electrodes placed 1 cm above and beneath the right eye respectively was used to detect eye blinks, and one electrode placed 1 cm from the outer canthus of the left eye was used to record the horizontal electrooculogram (EOG). The online filtering range was 0.1~250 Hz, and an electrode placed at the left mastoid acted as the recording reference.

The EEG data were pre-processed and analyzed using the EEGlab toolbox (Version 13.3.2b^40^) and the ERPlab plugin^41^ for Matlab (The Math works Inc., Natick, Massachusetts, USA). Trials with excessive EEG artifacts were excluded by visual inspection. The ERP epochs were trimmed (from 200 ms before the presentation of the WM load stimuli to 1000 ms thereafter) and the pre-stimulus baseline (−200 to 0 ms) were corrected. The EEG signals were re-referenced to the averaged mastoid electrodes during the offline processing. The ocular artifacts were removed using independent component analysis (ICA, runica algorithm). The data were band-pass filtered from 0.01 to 30 Hz and those epochs with amplitudes exceeding ±75 μV were excluded. On average, 56.77 trials (range: 30~64) of the artifact-free ERPs were averaged for each of the six experimental conditions (3 (emotion: fear, disgust, neutral) × 2 (WM load: low, high)) for each participant for further analysis.

### Statistical Analysis

The statistical analysis was conducted in ez package built in R version 3.3.2. For the behavioral data, repeated measures analysis of variances (ANOVA) were performed separately on the reaction time (RT), accuracy (ACC)with Group (HDs, CSs) as a between-subject factor, Emotion (neutral, fear and disgust) and WM Load (low, high) as within-subject factors. Trials with incorrect responses were excluded from the RT analysis.

For the ERP data, repeated measures ANOVAs were performed on the mean amplitudes of each ERP component with Channels as an additional within-subject factor. A significant interaction was followed by post-hoc ANOVAs to examine simple effects. Degrees of freedom for F-ratios were corrected according to the Greenhouse-Geisser method. Statistical differences were considered significant at *p* < 0.05; post-hoc comparisons were Bonferroni-corrected at *p* < 0.05.

## Results

### Behavioral Results

A 2 (group) × 3 (emotion) × (WM load) ANOVA performed on RT revealed a significant main effect of emotion (*F* = 8.49, *p* < 0.001) and a significant main effect of WM load (*F* = 362.83, *p* < 0.001). Pairwise comparison revealed a significantly longer RT for neutral than for fear and disgust (*p*s < 0.001) but the difference between fear and disgust was insignificant.

**Figure 2:**
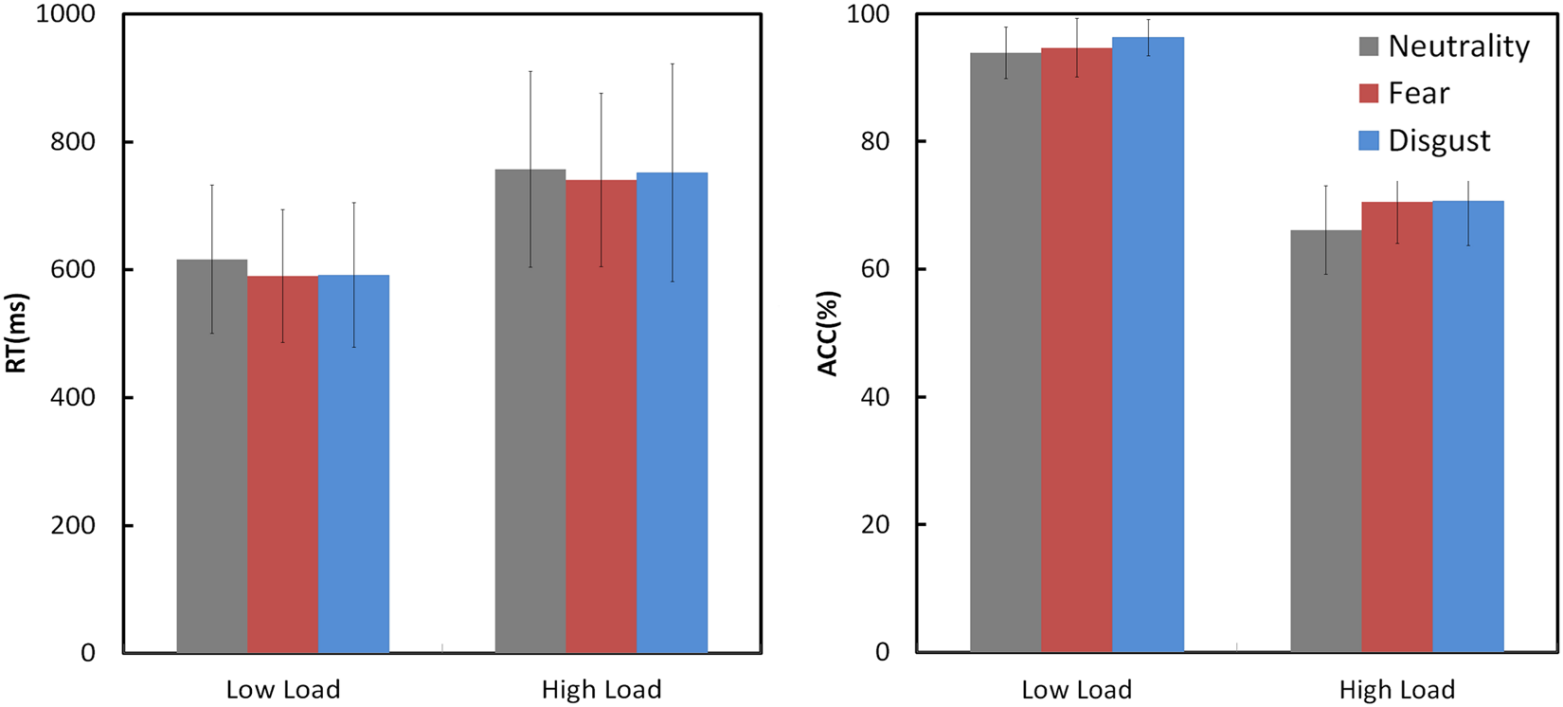
The histogram of behavioral results. RT = reaction time; ACC = accuracy.

A 2 (group) × 3 (emotion) × 2 (WM load) ANOVA performed on ACC revealed a significant interaction of Emotion × WM load (*F* = 3.48, *p* = 0.037). The post-hoc ANOVAs revealed that in both of the High (*F* = 12.78, *p* < 0.001) and Low WM load (*F* = 8.84, *p* < 0.001) conditions the ACC of neutral expressions was significantly lower than the ACCs of disgust and fear (*p*s < 0.014). No other main effect or interactions were found significant (*p*s > 0.204). Details of the behavioral measurements were presented in Table 2 and Fig. 2.

**Table 2:** Behavioral Measures for Hazardous Drinkers (HDs) and Control Subjects (CSs) in Each Experimental Condition of Emotional Working Memory Task: Mean (SD).

	Group	Low Load			High Load		
		Neutrality	Fear	Disgust	Neutrality	Fear	Disgust
RT	HDs	617 (100)	605 (111)	604 (103)	746 (141)	752 (155)	758 (162)
	CSs	615 (132)	575 (97)	579 (123)	768 (166)	729 (117)	745 (179)
ACC	HDs	93.6 (4.6)	94.8 (4.8)	96.8 (2.3)	64.9 (5.4)	69.9 (5.2)	70.0 (5.4)
	CSs	94.2 (3.5)	94.6 (4.4)	95.8 (3.4)	67.3 (8.5)	71.1 (7.8)	71.3 (8.6)

Note. RT = reaction time (ms); ACC = accuracy (%).

### ERP Results

**Figure 3:**
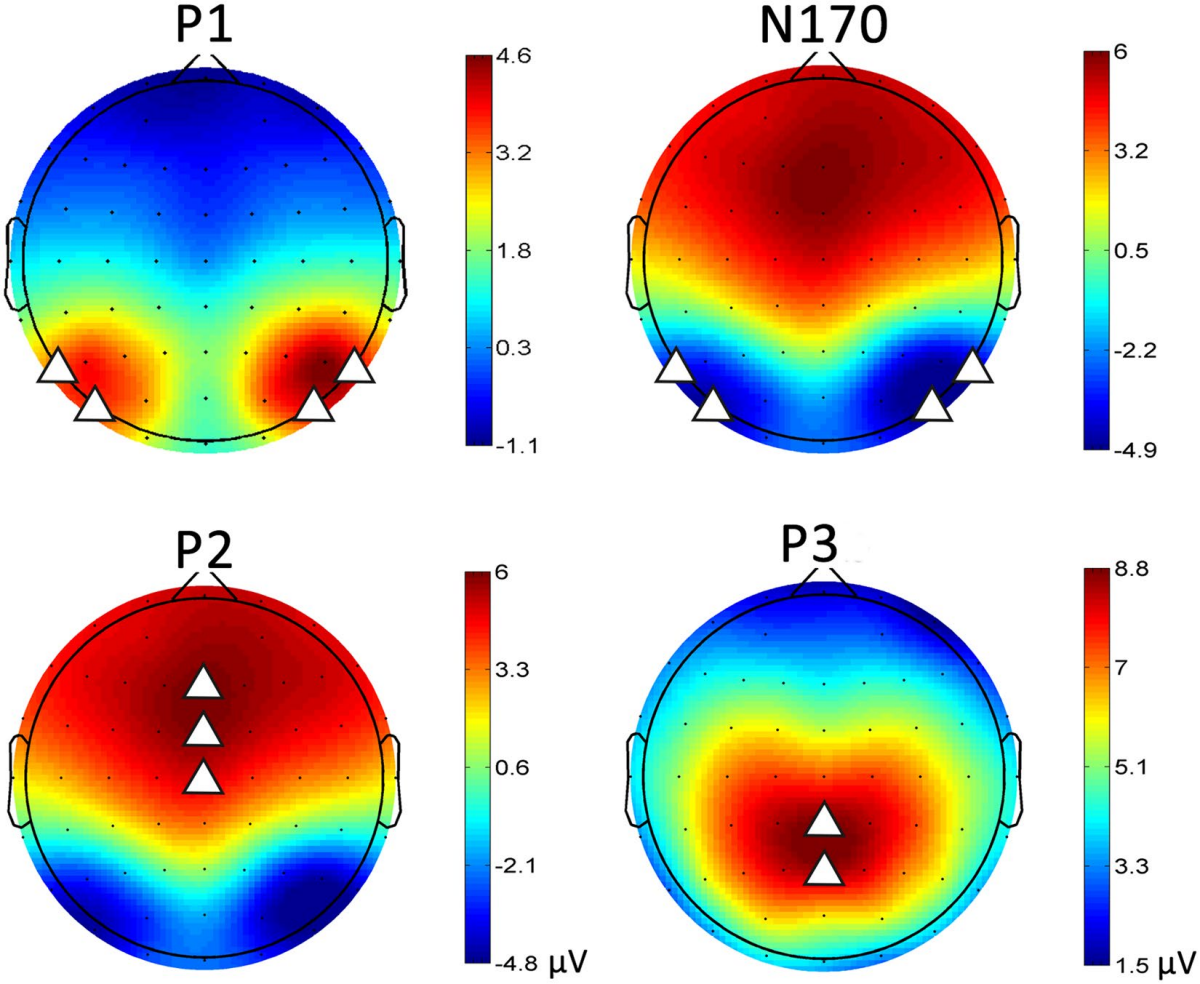
The topographies of the ERP components. The triangles in the topographic maps indicate the electrodes for analysis.

The present study focused on the ERPs elicited by facial expressions in the WM task. We analyzed the components of the P1 (100~130 ms) and N170 (150~200 ms) at lateral parietal occipital sites (PO7/PO8). Since P2 (150~200 ms) and P3 (350~500 ms) reached their maximum in the midline according to the grand-averaged ERP, we selected the average of Fz and FCz for the analysis for P2 and the average of Cz, CPz, and Pz for P3. The measurements of each component were illustrated in Table 3 and Fig. 3.

**Table 3:** ERP Amplitudes and Latencies for Hazardous Drinkers (HDs) and Control Subjects (CSs) in Each Experimental Condition of Emotional Working Memory Task: Mean (SD).

	Group	Low Load			High Load		
		Neutrality	Fear	Disgust	Neutrality	Fear	Disgust
*P1*							
Amp	HDs	4.6 (1.93)	4.35 (2.03)	4.54 (2.31)	4.4 (1.72)	4.37 (2.18)	4.16 (1.86)
	CSs	4.84 (2.82)	4.75 (2.69)	4.86 (2.71)	4.61 (2.49)	4.58 (2.45)	4.9 (2.33)
Lat	HDs	114 (13)	110 (14)	114 (13)	110 (17)	113 (13)	113 (13)
	CSs	111 (15)	109 (13)	111 (12)	111 (14)	108 (14)	109 (16)
*N170*							
Amp	HDs	−5.25 (3.84)	−5.29 (4.22)	−5.08 (3.87)	−5.4 (3.88)	−5.22 (4.37)	−5.56 (3.92)
	CSs	−5.46 (3.63)	−5.31 (3.47)	−5.41 (3.24)	−5.99 (3.38)	−5.3 (2.99)	−5.96 (3.55)
Lat	HDs	180 (11)	180 (13)	178 (12)	178 (13)	179 (13)	179 (12)
	CSs	172 (12)	174 (11)	172 (12)	171 (12)	172 (12)	172 (12)
*P2*							
Amp	HDs	6.89 (3.71)	6.11 (3.65)	6.03 (3.07)	7.85 (3.47)	8.38 (3.33)	7.09 (3.14)
	CSs	5.48 (2.68)	5.26 (2.68)	6.1 (2.72)	6.74 (2.67)	6.57 (2.53)	6.55 (2.57)
Lat	HDs	178 (14)	178 (13)	180 (13)	179 (14)	180 (14)	182 (14)
	CSs	172 (13)	176 (12)	175 (13)	176 (12)	181 (14)	178 (11)
*P3*							
Amp	HDs	9.87 (3.78)	10.68 (3.31)	11.25 (3.34)	8.88 (3.57)	9.51 (3.27)	9.19 (3.76)
	CSs	11.26 (2.95)	12.14 (3.71)	11.23 (3.35)	10.15 (2.56)	10.12 (3.03)	9.92 (2.74)
Lat	HDs	423 (43)	444 (45)	427 (55)	406 (66)	416 (64)	412 (63)
	CSs	429 (57)	431 (58)	445 (63)	394 (47)	385 (45)	390 (52)

Note. Amp = Amplitude (µV); Lat = Latency (ms).

#### P1

**Figure 4:**
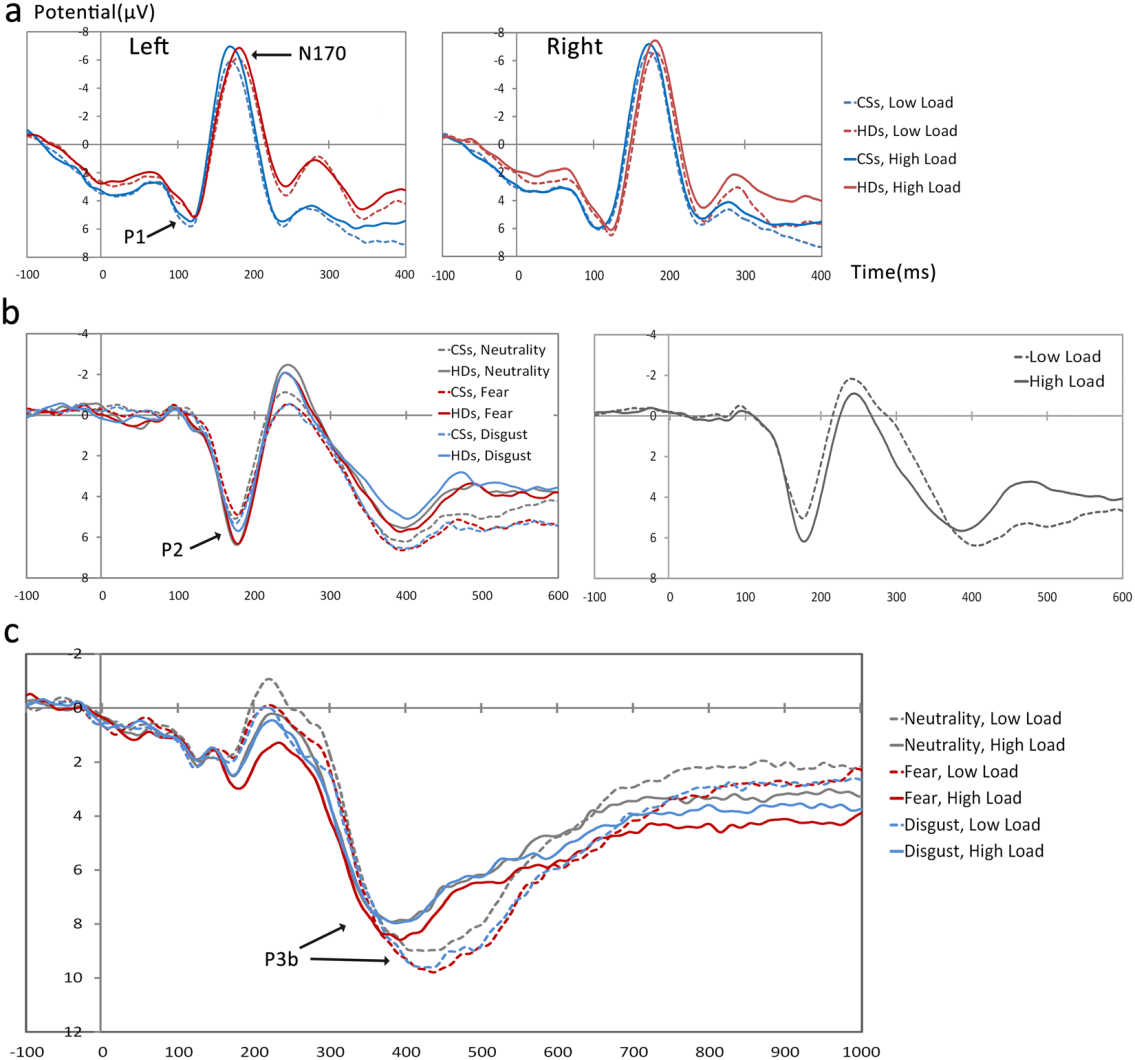
The waveforms of (**a**) P1 and N170 at parietal-occipital sites, (**b**) P2 at frontal-central sites and (**c**) P3 at central-parietal sites.

A 2 (group) × 3 (emotion) × 2 (WM load) × 2 (channel: PO7 and PO8) ANOVA performed for the peak amplitude and latency of P1. For the latency, a significant interaction of emotion × group × channel (*F* = 3.77, *p* = 0.028, *η* ^*2*^ = 0.11) was observed. The post-hoc ANOVAs showed the main effect of group under the fear condition at the PO8 site was significant with longer P1 latency in the HDs compared with the CSs (115 ms vs. 106 ms)(Fig. 4a).

#### N170

A 2 (group) × 3 (emotion) × 2 (WM load) × 2 (channel: PO7 and PO8) ANOVA was performed for the peak amplitude and latency of N170. For the amplitude, a significant main effect of WM load with larger peak amplitude (more negative) for High WM load compared to the Low WM load (−5.66 µV vs. −5.10 µV, *F* = 19.94, *p* < 0.001, *η* ^*2*^ = 0.35) was revealed. For the latency, we found a marginally significant main effect of group with the HDs showing longer N170 latency than the CSs (179 ms vs. 172 ms, *F* = 3.95, *p* = 0.056, *η* ^*2*^ = 0.11)(Fig. 4a).

#### P2

2 (group) × 3 (emotion) × 2 (WM load) ANOVAs were performed for the mean amplitude and latency of P2. For the amplitude, we found a significant main effect of WM load with enhanced P2 amplitude for high WM load compared to the low WM load (7.20 µV vs. 5.98 µV, *F* = 27.10, *p* < 0.001, *η* ^*2*^ = 0.47). There was also a significant interaction between of emotion × WM load (*F* = 4.09, *p* = 0.021, *η* ^*2*^ = 0.12). The post-hoc ANOVAs showed a significant simple effect of WM load for all of the emotion conditions while no significant simple effect of emotion was found for neither of the WM load conditions. Besides, a significant interaction of emotion × group was also revealed (*F* = 3.60, *p* = 0.033, *η* ^*2*^ = 0.10); the simple effect of emotion was found in the HDs with higher amplitudes for the neutral/fear compared with disgust conditions (7.37 µV/7.24 µV vs. 6.56 µV), which was not significant in the CSs. For the latency, the analysis revealed a significant main effect of emotion (*F* = 4.90, *p* = 0.011, *η* ^*2*^ = 0.14), which could be further explained by an interaction of emotion × group (*F* = 2.77, *p* = 0.071, *η* ^*2*^ = 0.08). The simple effect analysis showed a significant main effect of emotion in CSs with shorter P2 latency for neutral compared with fear/disgust conditions (174 ms vs. 179/177 ms, *F* = 6.02, *p* = 0.006, *η* ^*2*^ = 0.27). However, this main effect of emotion was not significant in the HDs. There was also a significant main effect of WM load, revealing longer P2 latency for high WM load compared to the low WM load (179 ms vs. 176 ms, *F* = 7.27, *p* = 0.011, *η* ^*2*^ = 0.19)(Fig. 4b).

#### P3

2 (group) × 3 (emotion) × 2 (WM load) ANOVA performed for the mean amplitude and latency of P3. For the amplitude, a significant main effect of WM load was observed, indicating an enhanced P3 for low WM load compared to the high WM load (11.07 µV vs. 6.93 µV, *F* = 33.93, *p* < 0.001, *η* ^*2*^ = 0.52). No other main effect or interactions were found significant. For the latency, the analysis revealed a main effect of WM load, exhibiting shorter P3 latency for high WM load relative to low WM load (401 ms vs. 433 ms, *F* = 24.01, *p* < 0.001, *η* ^*2*^ = 0.44)(Fig. 4c). Neither main effect nor any other interactions were significant.

## Discussion

The aim of the present study was to examine whether the cognitive functions related to emotional WM were disrupted in the HDs. ERP data provided evidences that the HDs and the CSs differ with respect to their electrophysiological responses to emotional stimulus during the WM processes. That is, despite preserved P3and comparable behavioral performance, the HDs showed abnormal neuro-electrophysiological activity indexed by P1, N170, and P2inthe early stage of emotional facial expression processing.

Modulated by spatial attention, visual P1 is elicited by low-level sensory processing at the visual extrastriate areas^21,22^. A previous study using the same paradigm but body expression as stimuli suggested that P1 was sensitive to WM load with shorter latencies and increasing amplitude with enhanced WM load^42^. In the present study, the longer P1 peak latency in the HDs indicates a delayed early low-level perception processing comparing to the CSs. This finding is compatible with that of Maurage *et al*. (2008), in which alcoholism induced longer P1 latency in a gender/expression identification task^31^. However, the group effect in our study was only found for fear, but not for neutral and disgust conditions. When it comes to neutral/disgust, the HDs and the CSs showed no significant difference. Some researchers suggested that the code differentiating threat and non-threat is partially stored in the perception system^43,44^, thus being less sensitive to process low-level cues of the expression of fear might indicate the abnormal activation of this decoding activity in the HDs.

N170 is regarded as a biomarker of face-specific processes driven by the high-level visual processes and the low-level stimulus properties^45^. In Morgan *et al*. (2008) study, N170 was found to be increased as the number of faces increased, and this N170 increase varied according to WM capacity. And the author suggested that this modulation of N170 by WM load showed that WM capacity limits are reflected in early stages of face processing^38^. In the present study, the tendency that the HDs had delayed N170 relative to the CSs indicates that they might have problems in detecting and encoding faces. This result is in line with the previous findings that showed a difference between the ADs and the CSs in the latency of N170^31^, indicating the speed for facial structural encoding process is slowed in the HDs.

On P2 component, we found an emotion × group interaction in both of the latency and the peak amplitude. It has been reported that P2 increased for incongruent relative to congruent targets, thereby the amplitude of P2 may indicate an enhanced cognitive processing demands^46^, and this was compatible with the WM load effect observed in the current study (*i.e*., elevated P2 in response to high WM load). Besides, HDs were found to have a reduced P2 for disgust compared to neutral/fear while this emotional effect was not found in the CSs. As P2 is influenced by the perceptual features of stimulus^27^ and increasing P2 at the frontal sites might reflect participants’ excessive engagement in feature detection^47^, this result may imply a reduced engagement to encode the expression of disgust in the HDs. Because the processing of disgust is dependent on the function of insula, the result of P2 is in accordance with that of the study by O’Daly *et al*. (2012), in which the alcoholics showed reduced activation in the insula as well as reduced connectivity between insula and the frontal cortex^48^. On the other hand, the latency of P2 was longer for the two negative expressions compared with neutral in the CSs, but this effect was not found in the HDs. This finding indicates that the HDs may have accelerated bottom-up processing triggered by the negative facial expressions. Further investigations are warranted to support these speculations.

It was suggested that the P3 reflected the conscious evaluation processes driven by top-down control for memory encoding^30^. This ability was found to be preserved in the HDs since no group difference was found either in the latency or the amplitude in the present dataset. Although the previous studies have demonstrated impaired P3/top-down control in the ADs^49^, this impairment is not obvious in those at risk for developing AD (*i.e*., HDs).Besides, the reduced P3 observed due to increasing task processing demands (*i.e*., reduced P3 in the high WM load than the low WM load conditions) was in concordance with a prior study which showed a suppressed P3 in increased WM load^50^. The P3 result is also compatible with the behavioral result, in which the HDs and the CSs had similar performance, indicating the probably preserved WM ability in the HDs.

The results of the current study provide the first evidence that the HDs might have delayed cognitive functioning at the early perception stage. They have impaired bottom-up processing triggered by emotional facial expressions, which needs further investigations. Thus the delayed perceptual process could be an indication of damage to cognitive functions caused by alcohol intoxication in the early stage. The WM ability, however, is relatively preserved in the HDs but at risk for being impaired if the drinking problems get worse (*e.g*., developing alcohol dependence)^11^.
